# The Post-Discharge Network Coordination Programme: A Randomized Controlled Trial to Evaluate the Efficacy of an Intervention Aimed at Reducing Rehospitalizations and Improving Mental Health

**DOI:** 10.3389/fpsyt.2016.00027

**Published:** 2016-03-03

**Authors:** Michael P. Hengartner, Silvia Passalacqua, Gisela Heim, Andreas Andreae, Wulf Rössler, Agnes von Wyl

**Affiliations:** ^1^Department of Applied Psychology, Zurich University of Applied Sciences (ZHAW), Zurich, Switzerland; ^2^Integrated Psychiatric Clinic of Winterthur and Zurich Unterland (ipw), Winterthur, Switzerland; ^3^Department of Psychiatry, Psychotherapy and Psychosomatics, University of Zurich, Zurich, Switzerland; ^4^Laboratory of Neuroscience (LIM27), Institute of Psychiatry, University of São Paulo, São Paulo, Brazil

**Keywords:** randomized controlled trial, rehospitalization, discharge, community care, case management, social work

## Abstract

**Purpose:**

To evaluate the efficacy of a post-discharge intervention for psychiatric inpatients aimed at preventing hospital readmissions and at improving patients’ mental health and psychosocial functioning.

**Methods:**

Randomized controlled trial using parallel group block randomization including 151 patients with ≤3 hospitalizations within the last 3 years, a GAF score ≤60, and aged 18–64 years, assessed at two psychiatric hospitals from the canton of Zurich, Switzerland, between September 2011 and February 2014. Primary outcomes were rate and duration of rehospitalization; secondary outcomes were mental health and functioning. Outcome measures were assessed before discharge from the index hospitalization (*t*_0_), 3 months after discharge when the intervention terminated (*t*_1_), and 12 months after discharge (*t*_2_). Participants received either a brief case management post-discharge intervention or treatment as usual.

**Results:**

In the short-term (i.e., *t*_0_–*t*_1_), no significant effect emerged in any outcome. In the long term (i.e., *t*_0_–*t*_2_), the two groups did not differ significantly with respect to the rate and duration of rehospitalization. Also, the intervention did not reduce psychiatric symptoms, did not improve social support, and did not improve quality of life. However, it did slightly increase assessor-rated general (*d* = 0.30) and social functioning (*d* = 0.42), although self-reports revealed a deteriorative effect on symptom remission (*d* = −0.44).

**Conclusion:**

This psychosocial post-discharge intervention showed no efficacy in the primary outcome of rehospitalization. With respect to secondary outcomes, in the long term it might lead to slightly increased social functioning but revealed no significant effect on psychopathology, social support, and quality of life. By contrast, with respect to self-reported symptom remission, it was revealed to have a negative effect. In this high-resource catchment area with comprehensive community psychiatric and social services, the intervention thus cannot be recommended for implementation in routine care.

## Introduction

The reduction of costly rehospitalization rates and duration of inpatient treatments constitutes a major objective of modern deinstitutionalized community mental health care in areas with high levels of resources (1). The time immediately after hospital discharge and the transition from inpatient to outpatient treatment is a pivotal time period for psychiatric patients, characterized by high risk of suicide and self-harm (2, 3). Rehospitalization is frequent because, unfortunately, many persons with mental disorders do not comply with appointments in outpatient services (4, 5), do not adhere to medication (6, 7), or disengage from outpatient care (8, 9). These findings emphasize the need for a rigorously planned and coordinated transition from inpatient to outpatient care and for continuity of care (10).

A systematic review of interventions aimed at reducing rates of readmission conducted by Vigod et al. (11) found a statistically significant effect of moderate to large magnitude in only 7 out of 15 studies. Steffen et al. (12), in their systematic review of 11 studies on discharge planning interventions after inpatient treatment, found a modest reduction in readmission rates and mental health problems as well as an increase of adherence to outpatient treatment, but not an improvement in quality of life. Moreover, the validity of those results was limited by the small number of trials and their small sample sizes and conclusions were mostly restricted to the USA. A recent multicentre randomized controlled trial (RCT) in Germany aimed at improving needs-oriented discharge planning [not included in Vigod et al. (11)] failed to find statistically and clinically significant effects (13). In line with this, meta-analyses of case management programs similarly produced mixed results and demonstrated that overall the effectiveness of case management is rather modest (14, 15). Thus, the questions as to whether there is any need for post-discharge interventions or whether a different approach should be adopted remain to be answered.

The aim of this RCT was to evaluate the effectiveness of a newly designed psychosocial post-discharge intervention named Post-Discharge Network Coordination Programme (PDNC-P). This intervention is in line with an emerging focus on resource-oriented therapeutic interventions that aim at fostering interpersonal relationships and social networks (16). As detailed in the study protocol (17), we specifically hypothesized that the PDNC-P would (a) reduce the rate and duration of rehospitalization, (b) reduce psychiatric symptoms, (c) improve social support, (d) improve quality of life, and (e) increase social functioning.

## Method

### Participants and Design

This study was conducted as part of the Zurich Programme for Sustainable Development of Mental Health Services (ZInEP; in German: “Zürcher Impulsprogramm zur nachhaltigen Entwicklung der Psychiatrie”), a research and health care program involving several psychiatric research divisions and mental health services from the canton of Zurich, Switzerland. This RCT initially included 167 participants from the Winterthur – Zurich Unterland psychiatric catchment area, an urban/suburban area of high level resources near the city of Zurich, Switzerland. The sample size was determined according to *a priori* calculations as detailed in von Wyl et al. (17), which assumed an expected medium effect size and a drop-out rate of 25%. The participants were enrolled at two different psychiatric hospitals, that is, the Psychiatrie-Zentrum Hard in Embrach and the Klinik Schlosstal in Winterthur, which are both part of the umbrella organization Integrierte Psychiatrie Winterthur – Zürcher Unterland (IPW). The inclusion criteria were as follows: (1) no more than three hospitalizations within the last 3 years (including the index hospitalization), (2) a Global Assessment of Functioning (GAF) score of 60 or lower, (3) cognitive ability to provide written informed consent, and (4) age between 18 and 64 years. Exclusion criteria were as follows: (1) insufficient German language proficiency, (2) simultaneous support by another case manager, and (3) patient living in supportive housing. Of the 167 randomized participants, 151 patients (90.4%) were included in the analysis. The 16 participants who were excluded from the analysis after the group allocation comprised cases that subsequently conflicted with the inclusion criteria (mainly because they received additional case management or were accommodated in supportive housing over the course of the study). Data analysis was conducted according to the logic of the intention-to-treat (18). The study was approved by the cantonal ethics committee of Zurich (reference number: KEK-ZH 2011-0175). The trial was registered in the International Standard Randomised Controlled Trial Number (ISRCTN) register (reference number: ISRCTN58280620) and the study protocol published and freely available online (17). This report was drafted according to the CONSORT statement (19).

### Randomization and Procedure

Participants were allocated randomly to either the intervention or control group with a stratified block randomization for the psychiatric diagnoses according to ICD-10 (20). The random allocation sequence was generated with Microsoft Excel and was implemented by a research associate who was not part of the study group. The intervention, named Post-Discharge Network Coordination Programme (PDNC-P), was developed in collaboration between the IPW and the Zurich University of Applied Sciences (ZHAW). The intervention program aims to improve hospital discharge planning and to ease the transition from inpatient to outpatient care by coordinating a social support network (21). The intervention was provided by two experienced social workers, to one of whom each patient from the intervention group was assigned. Each patient met his social worker prior to discharge and collaboratively agreed upon a close network of social support, a crisis plan, and the terms of program termination. After discharge, a close person from the patient’s social network was assigned to be network representative. Also, mostly after discharge, the social workers were instructed to organize an interdisciplinary care review meeting that included the most important persons from the network (in some cases, the meeting took place before discharge). The social worker then visited the patient within the first week after discharge to support and monitor the patient’s adjustment to outpatient care and daily life. After the first-week home visit, the social worker scheduled subsequent visits. The program was tailored to meet the patient’s personal needs and the frequency of the visits was based on the patient’s personal progress. The intervention was directly targeted at promoting recovery through social relationships, which is a key element of resource-oriented therapies (16). The intervention concluded once the terms of termination were reached or after a maximum of 3 months post-discharge from inpatient care (i.e., at *t*_1_). Afterwards the social support network continued to aid the patient without the social worker’s assistance. For a detailed rationale of the intervention program, see Hengartner et al. (21).

The control group received treatment as usual, which in Switzerland comprises the patient receiving assistance from a social worker during his or her inpatient stay only if prescribed by the treating physician. Any support by the hospital’s social worker ends when the patient is discharged from hospital. However, after discharge some patients still see social workers who are not affiliated with a psychiatric hospital, but instead with the social welfare office of a larger urban community or psychiatric outpatient services. Therefore, patients in the control group might also have seen a social worker during the intervention period, depending on their individual needs.

**Figure 1:**
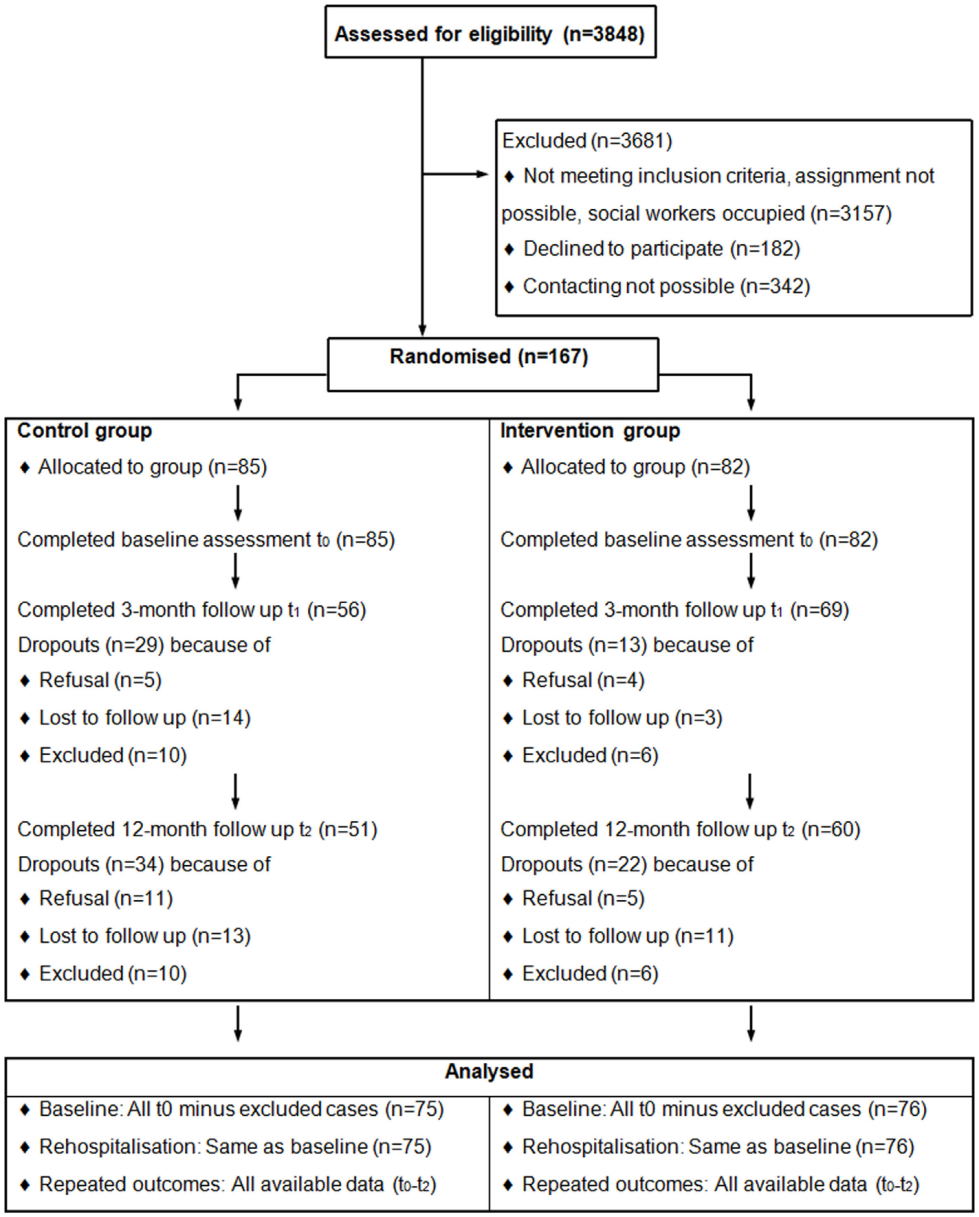
**Participants flow-chart**.

Both groups were assessed prior to discharge from the index hospitalization (*t*_0_), 3 months after discharge when the intervention terminated (*t*_1_), and 12 months after discharge (*t*_2_). Participants and evaluators were blind to their group allocation at baseline measurement *t*_0_ only, because masking was not feasible once the intervention had started. The recruitment began in September 2011 and the last follow-up assessment of *t*_2_ was carried out in April 2015. The participants’ flow is indicated in Figure 1.

### Outcome Measures

All instruments and measures applied in this study are extensively researched and widely applied in clinical practice and have all shown good reliability and validity. Because of space limits imposed by the journal, we therefore refrain from providing detailed information here and refer to the scientific literature or to von Wyl et al. (17). In short:

Primary outcome: the frequency of readmissions and the duration of inpatient stays were assessed using the IPW clinical registry and the Client Socio-Demographic and Service Receipt Inventory – European Version (CSSRI-EU) (22).

Secondary outcomes: patients’ social functioning was measured with the Social and Occupational Assessment Scale (SOFAS) (23). Global functioning was assessed using the GAF scale (24). Both GAF and SOFAS are administered by clinicians and range from 1 (extremely impaired functioning) to 100 (excellent functioning). Social support was measured with the Fragebogen zur sozialen Unterstützung – Kurzform 14 (F-SozU K-14) (25). The F-SozU K-14 is a German self-rating questionnaire, comprising items from the following three domains of perceived social support: emotional support, instrumental support, and social integration. Psychopathological distress and illness severity was assessed with an assessor-rated scale, the Health of the Nation Outcome Scales (HoNOS) (26), as well as with one self-rating instrument, the Outcome Questionnaire (OQ-45, German version) (27). Finally, quality of life was captured with the self-rating scale Manchester Short Assessment of Quality of Life (MANSA) (28).

### Statistical Analysis

The group allocation variable (control vs. intervention) was included as the independent or predictor variable in all models. The distribution of various measures across groups at *t*_0_ was analyzed with independent samples Mann–Whitney U tests for continuous variables and with contingency tables and χ^2^ tests for categorical variables. Number of rehospitalizations and inpatient days were analyzed with generalized linear models using Poisson distribution and log-link function. For rehospitalization when defined as a dichotomous outcome (no vs. yes), we fitted a binomial logistic regression model. The repeated measures of all outcomes over time in relation to group were examined with a series of generalized estimating equations (GEE) (29). These models were introduced to fit regression analyses that account for within-subject correlation, which is an inherent part of longitudinal studies that rely on repeated measures. Owing to the probability density function of the dependent variables, a Gamma distribution with log-link function best fitted our data for all outcomes (i.e., HoNOS, GAF, SOFAS, F-SozU, MANSA, and OQ-45). Since the total score of the F-SozU was originally left skewed, it was inverted for statistical analysis in order to change its distribution from left skewed to right skewed. As a result, after transformation higher scores indicate less social support. The within-subject covariance was specified with the “unstructured” correlation type to avoid any constraints on the covariance structure. A robust estimator was used to reduce the effects of outliers and influential observations. The intercept and slope factor were included in all analyses, which is standard procedure in longitudinal data modeling (30). In longitudinal analyses, the intercept corresponds to the baseline value of the repeated measures and the slope corresponds to the linear growth rate of those measures (i.e., time-trend). In addition to adjust for the within-subject correlation, the slope factor was also modeled as an interaction effect with the group variable to examine changes in the outcomes over time in relation to group allocation (i.e., intervention*time). The advantage of such a modeling approach is that its estimates are independent of group differences in baseline values (30). The interaction term was modeled in two different ways: once from *t*_0_ to *t*_2_ to examine group differences across study onset and 12-month follow-up (i.e., enduring effect) and once from *t*_0_ to *t*_1_ to examine group differences across study onset and termination of the intervention at 3-month follow-up (i.e., immediate effect). All analyses were conducted with SPSS 21 for Windows.

## Results

The baseline demographic and clinical characteristics at *t*_0_ are shown in Table 1. Scores on the HoNOS were on average slightly higher in the intervention group (*p* = 0.007). However, the corresponding effect size was small (Cohen’s *d* < 0.3). The distribution of all other variables did not vary significantly across groups (all *p* > 0.05).

**Table 1:** **Baseline descriptive statistics (*t*_0_)**.

		Group		Test statistic	*p*
		Control (*n* = 75)	Intervention (*n* = 76)		
Age	Years (SD)	41.0 (11.3)	42.1 (11.4)	*U* = 2954.0	0.699
Sex	Men (%)	39 (49.4)	40 (50.6)	χ^2^ = 0.0 (df = 1)	0.938
	Women (%)	36 (50.0)	36 (50.0)		
Marital status	Single (%)	31 (49.2)	32 (50.8)	χ^2^ = 0.4 (df = 2)	0.809
	Partnership/married (%)	18 (46.2)	21 (53.8)		
	Sep./div./widowed (%)	26 (53.1)	23 (46.9)		
Education level	Low (%)	16 (47.1)	18 (52.9)	χ^2^ = 0.1 (df = 2)	0.933
	Moderate (%)	41 (50.0)	41 (50.0)		
	High (%)	18 (51.4)	17 (48.6)		
Present hospitalization^a^	First (%)	43 (50.6)	42 (49.4)	χ^2^ = 4.1 (df = 2)	0.126
	Second (%)	18 (40.0)	27 (60.0)		
	Third (%)	14 (66.7)	7 (33.3)		
Primary diagnosis	SUD (%)	19 (51.4)	18 (48.6)	χ^2^ = 0.7 (df = 3)	0.862
	Psychosis (%)	22 (53.7)	19 (46.3)		
	Mood disorder (%)	25 (48.1)	27 (51.9)		
	Others (%)	9 (42.9)	12 (57.1)		
HoNOS	Mean (SD)	16.03 (5.49)	18.64 (6.34)	*U* = 3578.5	0.007
GAF	Mean (SD)	36.84 (11.10)	34.30 (10.32)	*U* = 2438.5	0.125
SOFAS	Mean (SD)	43.24 (11.61)	40.26 (11.75)	*U* = 2401.0	0.094
F-SozU (inv.)	Mean (SD)	2.29 (0.91)	2.43 (0.91)	*U* = 3058.0	0.282
MANSA	Mean (SD)	4.43 (1.02)	4.19 (1.13)	*U* = 2462.5	0.189
OQ-45	Mean (SD)	74.02 (24.08)	73.66 (30.43)	*U* = 2389.5	0.918

*HoNOS, Health of the Nation Outcome Scales; GAF, Global Assessment of Functioning; SOFAS, Social and Occupational Assessment Scale; F-SozU (inv.), Fragebogen zur sozialen Unterstützung (inverted) [Social Support Questionnaire]; MANSA, Manchester Short Assessment of Quality of Life; OQ-45, Outcome Questionnaire 45*.

*^a^Refers to the past 3 years*.

Overall, the number of hospital readmissions at the 12-month follow-up ranged from 0 to maximally 6, with a mean number of 0.52 and a SD of 1.06. The total duration of rehospitalizations ranged from 0 to 191 days with a mean and SD of 12.99 and 29.41. The two measures did not differ significantly between groups (both *p* > 0.65 and *d* < 0.2) (see Table 2). Adjustment for sex and age did not alter the results; both covariates did not relate to rehospitalization rate and duration. We additionally examined the total number of outpatient visits according to self-reports from the CSSRI-EU. Those numbers did not differ significantly either (*M* intervention = 13.67; *M* control = 10.48; Wald χ^2^ = 2.30, df = 1, *p* = 0.121). Here, a main effect for sex was found, with women reporting significantly more outpatient visits (women: mean visits [95% CI] = 15.26 [12.67–18.39]; men: mean visits = 8.90 [6.60–12.99]; *p* = 0.001). However, there was no interaction effect between treatment arm and sex (*p* = 0.522).

**Table 2:** **Number of rehospitalizations and total inpatient days at 12-month follow-up according to clinical registry records (primary outcomes)**.

	Group	Mean	95% CI	Test statistic	*p*
Rehospitalizations	Intervention	0.55	0.37; 0.84	χ^2^ = 0.17 (df = 1)	0.677
	Control	0.48	0.29; 0.81		
Inpatient days	Intervention	12.96	8.44; 19.91	χ^2^ = 0.00 (df = 1)	0.991
	Control	13.01	7.29; 23.22		

**Figure 2:**
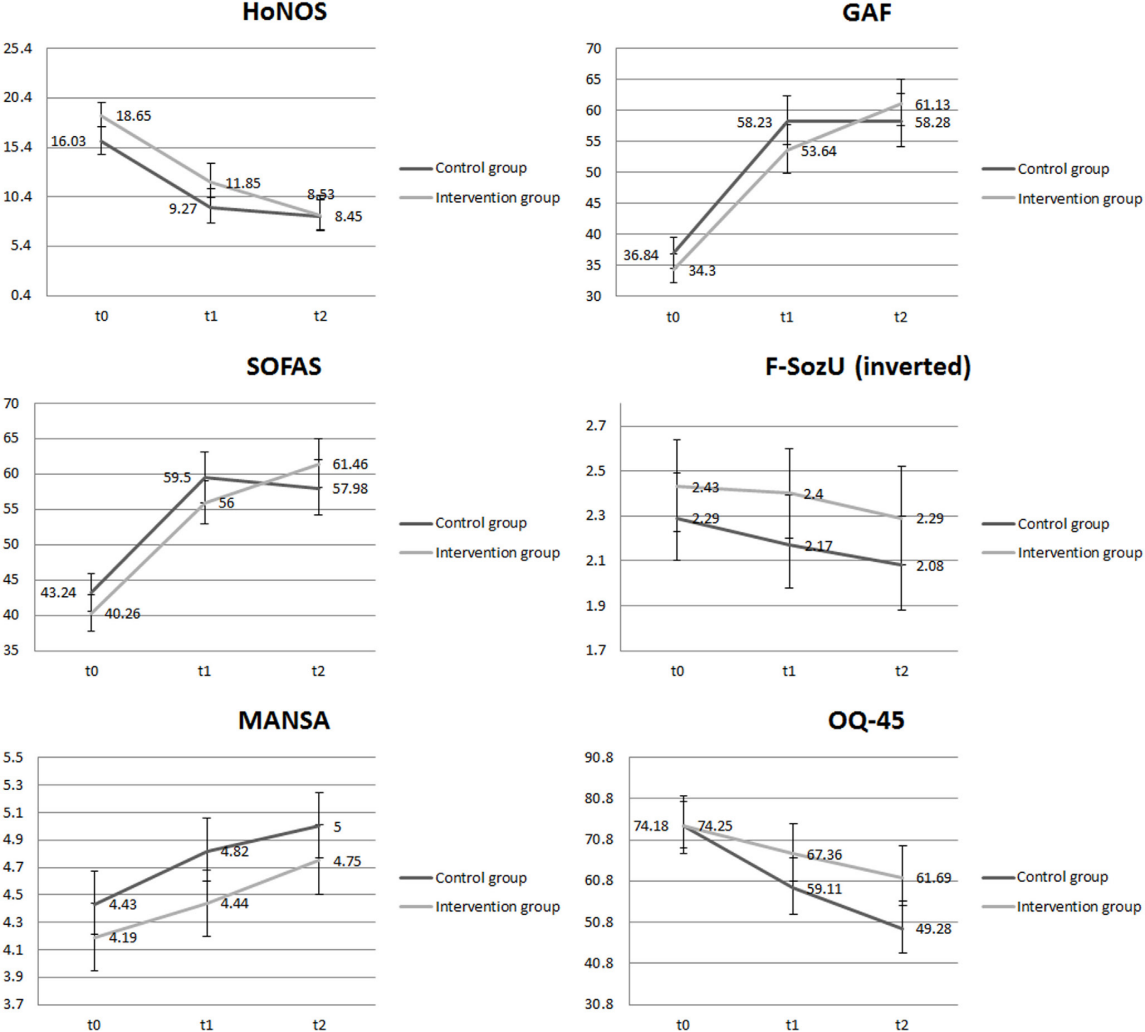
**Results of repeated outcome measures (secondary outcomes)**. HoNOS, Health of the Nation Outcome Scales; GAF, Global Assessment of Functioning; SOFAS, Social and Occupational Assessment Scale; F-SozU (inv.), Fragebogen zur sozialen Unterstützung (inverted) [Social Support Questionnaire]; MANSA, Manchester Short Assessment of Quality of Life; OQ-45, Outcome Questionnaire 45.

All other outcome measures were examined longitudinally with repeated measures. Their means and 95% confidence intervals are depicted graphically in Figure 2. The corresponding statistical significance testing of the regression coefficients using a series of GEE is shown in Table 3. We found no significant interaction between groups and trajectories from baseline to 3-month follow-up with respect to all outcomes (intervention*time *t*_0_, *t*_1_). However, values differed significantly from baseline to 12-month follow-up between groups (intervention*time *t*_0_, *t*_2_) with respect to GAF, SOFAS, and OQ-45. As measured with both GAF and SOFAS, patients in the intervention group showed slightly better functioning over time compared to those in the control group (Cohen’s *d* = 0.30 and 0.42, respectively). As for the OQ-45, participants in the control group had a steeper decline, indicating that their subjective distress improved more than those in the intervention group (Cohen’s *d* = −0.44).

**Table 3:** **Results of a series of GEE. Control group is the reference (secondary outcomes)**.

		*b*	95% CI	Test statistic	*p*
HoNOS	Intervention * Time *t*_0_, *t*_2_	−0.14	−0.40; 0.11	χ^2^ = 1.2 (df = 1)	0.278
	Intervention * Time *t*_0_, *t*_1_	0.10	−0.14; 0.33	χ^2^ = 0.6 (df = 1)	0.426
GAF	Intervention * Time *t*_0_, *t*_2_	0.12	0.01; 0.23	χ^2^ = 4.3 (df = 1)	0.040
	Intervention * Time *t*_0_, *t*_1_	−0.01	−0.14; 0.12	χ^2^ = 0.0 (df = 1)	0.869
SOFAS	Intervention * Time *t*_0_, *t*_2_	0.13	0.02; 0.24	χ^2^ = 5.1 (df = 1)	0.024
	Intervention * Time *t*_0_, *t*_1_	0.01	−0.10; 0.12	χ^2^ = 0.0 (df = 1)	0.843
F-SozU (inv)	Intervention * Time *t*_0_, *t*_2_	0.04	−0.09; 0.17	χ^2^ = 0.4 (df = 1)	0.543
	Intervention * Time *t*_0_, *t*_1_	0.04	−0.06; 0.15	χ^2^ = 0.6 (df = 1)	0.432
MANSA	Intervention * Time *t*_0_, *t*_2_	0.01	−0.06; 0.07	χ^2^ = 0.0 (df = 1)	0.891
	Intervention * Time *t*_0_, *t*_1_	−0.03	−0.11; 0.05	χ^2^ = 0.4 (df = 1)	0.507
OQ-45	Intervention * Time *t*_0_, *t*_2_	0.23	0.08; 0.37	χ^2^ = 8.9 (df = 1)	0.003
	Intervention * Time *t*_0_, *t*_1_	0.13	0.00; 0.26	χ^2^ = 3.8 (df = 1)	0.050

*HoNOS, Health of the Nation Outcome Scales; GAF, Global Assessment of Functioning; SOFAS, Social and Occupational Assessment Scale; F-SozU (inv.), Fragebogen zur sozialen Unterstützung (inverted) [Social Support Questionnaire]; MANSA, Manchester Short Assessment of Quality of Life; OQ-45, Outcome Questionnaire 45*.

## Discussion

### General Discussion

This RCT was conducted to evaluate a newly designed psychosocial intervention named Post-Discharge Network Coordination Programme (PDNC-P), which is a brief form of transitional case management. For more information, see Hengartner et al. (21). The PDNC-P was primarily conceived to reduce instant readmission after psychiatric hospitalization and secondarily to improve patients’ mental health, social support, quality of life, and social functioning. This intervention was designed according to the emerging recovery approach and its focus on the personal needs of the service user (31). As recently reviewed by Priebe et al. (16), social relationships form a key element in resource-oriented therapeutic interventions. Nevertheless, the intervention yielded no significant immediate short-term effect at all (i.e., 3-month follow-up). In the long term (i.e., 12-month follow-up) and with respect to both primary outcomes and most secondary outcomes, our program yielded no effect either. That is, the PDNC-P did (a) not reduce the rate and duration of rehospitalization, (b) did not reduce psychiatric symptoms, (c) did not improve social support, and (d) did not improve quality of life. However, (e) it slightly increased social functioning in the long term according to GAF and SOFAS, but on the other hand (f) with respect to self-reported illness severity (i.e., OQ-45), we found that the intervention had a moderate negative effect. That is, participants in the intervention group indicated significantly less symptom remission in the long term than participants in the control group. This is an unexpected finding that needs careful examination in further studies. Although it has been shown that some psychosocial interventions may cause harm in certain patients (32, 33), it would be premature to draw any conclusions on this issue here without additional analyses. Our main objective in future research will, thus, certainly be to conduct comprehensive in-depth analyses with respect to the OQ-45.

For the time being, in an attempt to integrate the findings of the present study with the literature, we conclude that they are mainly in line with the lack of association and inconsistent results of previous studies. For instance, a needs-oriented discharge planning across multiple sites in Germany evaluated by Puschner et al. (13) did not reveal any positive effect on the patients’ psychopathology and hospital readmission rates. Neither did, for instance, another well-known RCT (34) nor a matched case-control study (35) from the US. A recently conducted systematic review showed mixed and inconclusive results as well, pointing out that only seven of 15 studies (that is, less than half) found a significant reduction in readmission rates (11). In an earlier systematic review, Steffen et al. (12) showed that discharge planning interventions had only a small effect on mental health outcomes and no effect on quality of life. Finally, meta-analyses of psychiatric case management also yielded rather modest beneficial outcomes overall, especially in patients who are not high-frequency users (14, 15). Hitherto, engagement with health services proved to be the only consistently replicated positive outcome in intensive case management programs (36). Therefore, a conservative interpretation of the literature would be that to date there is no compelling evidence of a reliable and sustainable (post-) discharge intervention with unequivocal and substantial long-term benefits, especially in care settings that have already achieved low rates of hospitalizations and in patients with rather low use of inpatient treatments (14).

Since case management programs appear to be effective only in severely impaired “revolving-door” patients (14, 15), it could be that our sample was not impaired enough. The rather low rehospitalization rates point toward such an explanation. In addition, as discussed previously (21), a social network intervention, such as the PDNC-P, builds on the premise that patients possess an adequately robust social network, an assumption that is certainly violated in many patients. Finally, the PDNC-P is not targeted at stable internal patient characteristics; it aims at altering external structures, that is, the patient’s social environment. Hence, a patient’s personality, in particular trait neuroticism, which is a major predictor of service use (37–40), remains mostly unaffected by such an intervention and may, thus, undermine its effectivity. More specifically, persons scoring high on neuroticism are less resilient to the effects of stressful life events (41, 42), respond inadequately to psychosocial treatments (43, 44), and have, which is crucial for a social network intervention, often disruptive relationships and poor interpersonal resources (45, 46). That is, for these patients the social network is not a resource, but rather a problem area. As, for instance, detailed in Hengartner (37) and Tyrer (47), we therefore suggest that future interventions should opt to consider patients’ personality traits to improve sustainability and effectivity of psychiatric interventions. Finally, in Switzerland, a comprehensive care system that offers support and consulting to persons with mental health and social problems is provided not only by inpatient psychiatric services but also by social welfare departments and outpatient community services in larger urban communities. Those regulatory community services provide, among others, socio-legal support for tenancy issues, occupational resettlement programs upon unemployment, and psychiatric nursing. In particular in the urban region of Zurich, a comprehensive and highly specialized network of mental health services and care providers has been established, which also includes many private offices of psychotherapists and psychiatrists. However, in suburban and rural communities, outpatient mental health services are less frequent. In those parts of the catchment area, only social assistance and home care exist, but no specialized services. Nevertheless, in communities with high mental health resources, for initially low-frequency users with minor treatment needs, it is possible that additional post-discharge interventions do not provide a benefit to the established psychosocial care and support services.

### Limitations and Generalizability

The generalizability of the results is limited insofar as only low-frequency users were included (i.e., patients with no more than three hospitalizations within the last 3 years). We felt obliged to conceive the study in this way because we had experienced that chronic high-frequency users were not suitable for this kind of intervention. Moreover, only 151 patients out of 3848 persons (4.0%) who were initially assessed for eligibility were eventually included in the analysis. As a consequence, the representativity and generalizability of the study may be restricted. However, this is a general limitation inherent to most, if not all, research in this field. The systematic exclusion of most patients in RCT-research poses a serious problem to the relevance and validity of RCT-findings for general mental health practice in the community (48). Another limitation is that blinding was feasible only at *t*_0_. Afterwards, the patients, the social workers, and the assessors were aware of which group each patient was allocated to (open-label trial). In an attempt to minimize bias, we ensured that participants were not always rated by the same assessor. It is also important to note that self- and assessor-ratings capture differential aspects of the same person, which is why they are commonly only moderately correlated (49). Another limitation that needs to be addressed is the drop-out rate of 33.5%. No measure of mental health and functioning at baseline (*t*_0_) predicted subsequent drop-out at *t*_1_ or *t*_2_. However, the analysis showed that the drop-out rate differed considerably between groups (40.0% in the control group vs. 26.8% in the intervention group). This is relevant insofar as it has been argued that both harmful and successful interventions may yield higher drop-out rates (33). Therefore, we cannot exclude a respective potential bias.

In conclusion, in this RCT, a post-discharge intervention comprising a brief case management and network coordination provided by a social worker did not yield a statistically and practically significant effect on rates and duration of rehospitalizations (primary outcomes). In respect to secondary outcomes, the intervention did neither relate to quality of life and social support at 12-month follow-up after hospital discharge. The intervention did slightly increase global and social functioning, though. However, with respect to enduring self-reported mental health, the intervention even exerted a deteriorative effect on patients’ recovery. As a consequence, we feel compelled to state that in contrast to the assessor-rated social functioning, the intervention demonstrated a negative effect on the patients’ self-reported mental health in the long term. Taken together, without modifications this intervention, thus, cannot be considered appropriate and helpful for patients without a preceding history of frequent hospitalizations in a setting with high resources and diverse mental health and social services as implemented in the region of Zurich, Switzerland. We, therefore, contend that the development and implementation of further psychosocial post-discharge interventions should be subject to close scrutiny.

## Ethics Statement

The authors assert that all procedures contributing to this work comply with the ethical standards of the relevant national and institutional committees on human experimentation and with the Helsinki Declaration of 1975, as revised in 2008. The study was approved by the ethics committee of the canton of Zurich (KEK).

## Author Contributions

MH drafted the manuscript and conducted all statistical analyses. SP and GH participated in data collection and writing of the manuscript. AA and AW designed the study and participated in writing. WR designed the complete research program (ZInEP) and participated in writing of the manuscript. All authors critically revised the manuscript and approved the final version.

## Conflict of Interest Statement

The authors declare that the research was conducted in the absence of any commercial or financial relationships that could be construed as a potential conflict of interest.
